# Protective effects of naringenin and naringin in organ ischemia/reperfusion injuries: a comprehensive narrative review

**DOI:** 10.3389/fphar.2025.1701726

**Published:** 2026-01-12

**Authors:** Min Hou, Daiyan Wei, Yanshun Wang, Xiaojian Zhang, Zhiwei Yao

**Affiliations:** 1 The Affiliated Taian City Central Hospital of Qingdao University, Taian, China; 2 The First Clinical Medical College of Lanzhou University, Lanzhou, China; 3 Liaocheng Hospital of Traditional Chinese Medicine, Shandong, China

**Keywords:** flavonoids, ischemia/reperfusion injury, molecular mechanisms, oxidative stress, pharmacology

## Abstract

Ischemia/reperfusion injury (IRI) refers to a condition in which ischemia is followed by reperfusion, leading to an exacerbation of the initial tissue damage. Currently, there are no specific therapeutic methods for IRI. Phytochemicals from natural products have the potential to develop noble drugs for IRI. Naringenin (NGE) and naringin (NG) are natural dietary flavonoids derived from ethnobotanical plants in Southeast and South Asia. NGE and NG have a wide range of pharmacological properties, including antioxidant, anti-apoptotic, and anti-inflammatory effects. As research on NGE and NG deepens, it has been found that they protect against IRI. We first summarize plant species containing NGE and NG from Southeast and South Asia in this article. Then, we highlight recent advances in NGE and NG for treating IRI in the myocardium, brain, intestines, kidneys, retinal, liver, spinal cord, skeletal muscles, and testicles. We find that NGE and NG possess antioxidant, anti-inflammatory, anti-apoptotic, anti-endoplasmic reticulum stress, anti-ferroptosis, anti-pyroptosis, and autophagy regulatory properties that protect organs from IRI. In addition, NGE and NG alleviate organ IRI through certain signaling pathways, including nuclear factor-κB, nuclear factor erythroid 2-related factor 2, phosphatidylinositol 3-kinase/AKT, cyclic guanosine monophosphate–adenosine monophosphate synthase–stimulator of interferon genes, sirtuin (SIRT) 1/SIRT 3, and hypoxia-inducible factor-1α. Furthermore, we investigate the interactions between these signaling pathways and inflammation, oxidative stress, and programmed cell death. Nevertheless, NGE and NG still face challenges related to pharmacokinetic interactions, bioavailability, and clinical safety assessments. Further studies will be needed to verify their safety and efficacy in clinical settings.

## 1 Introduction

Ischemia/reperfusion injury (IRI) refers to the restoration of blood flow after ischemia and is one of the leading causes of death in ischemic diseases (Tian et al., 2024). IRI is a highly destructive pathological process present in various organs, such as the myocardium, brain, liver, lungs, kidneys, gastrointestinal tract, and retina (de Groot and Rauen, 2007). In addition to tissue damage, IRI can even trigger systemic inflammatory response syndrome and multiple organ dysfunction syndrome (Wu et al., 2018). IRI’s pathological mechanism is highly complex, and oxidative stress, inflammation, apoptosis, autophagy, and ferroptosis participate in the mechanism, forming an intricate network (Kalogeris et al., 2016; Turer and Hill, 2010). Different strategies have been developed for preventing and treating IRI, including physical therapies, gene therapies, drug therapies, and cell protection strategies (Li et al., 2025). At present, there is no specific drug for IRI owing to complex multifactorial damage and side effects. Consequently, novel agents with better efficacy and fewer side effects are needed to prevent IRI.

Increasingly, studies are focusing on natural products, including metabolites derived from plants (Ahmadi and Shadboorestan, 2016; Ahmadi et al., 2015). Southeast and South Asia are abundant in natural plant biodiversity, with a long history of medicinal plants (Ware et al., 2023). Naringenin (NGE) and its glycoside, naringin (NG), are natural dietary flavonoids found in citrus fruits (e.g., grapefruit and tomatoes) and ethnobotanical plants in Southeast and South Asia (Heidary Moghaddam et al., 2020; Stabrauskiene et al., 2022). Several studies have demonstrated that NGE and NG exhibit multiple biological properties, including antioxidant, anti-inflammatory, anti-ulcerative, and anticancer properties (Ahmadi and Shadboorestan, 2016). Meanwhile, multiple studies have demonstrated that NGE and NG improve IRI, including the myocardium, brain, intestines, kidneys, retina, liver, spinal cord, skeletal muscle, and testicles. Despite several reviews describing the role of NGE and NG in myocardial infarction (Kampa et al., 2023), brain ischemic (Chen et al., 2020), and acute kidney injury (Amini et al., 2022), to the best of our knowledge, the protective mechanisms of NGE and NG in multi-organ IRI have not been summarized. Integrated multi-organ studies can identify pathways that play protective roles across different organs, which may be overlooked in organ-specific research. This article reviews the latest preclinical evidence of NGE and NG on organ IRI, providing a more comprehensive and in-depth perspective on therapeutic strategies.

## 2 Isolation report of NGE and NG in Southeast and South Asian plants

Numerous natural products and medicinal plants are found in tropical and subtropical areas (particularly Southeast Asia and South Asia) (Prasad et al., 2016; Ware et al., 2023). Southeast Asia and South Asia have the highest levels of medicinal plant diversity due to their favorable environmental and geographical conditions (Prasad et al., 2016). There are several countries in the Southeast Asia region, including Indonesia, Thailand, Singapore, Cambodia, Malaysia, Laos, Vietnam, Timor-Lest, Myanmar, the Philippines, and Brunei. As the largest country in Southeast Asia, Indonesia is known for its endemism and rich diversity of species (Sun et al., 2024). Indonesia has 56%, 22%, and 8.7% of the world’s vascular plants in terms of families, genera, and species, respectively (Sun et al., 2024). As the second largest country in Southeast Asia, Myanmar’s rich biodiversity is well known, with an estimated 11,800 species of angiosperms and gymnosperms (Kyaw et al., 2021). Southern Asia has eight countries, namely, India, Afghanistan, Pakistan, Nepal, Bangladesh, Sri Lanka, Bhutan, and the Maldives (Mukherjee and Mackessy, 2021). India covers 2.4% of global land area and 8% of the world’s biodiversity (Gupta et al., 2022). More than 4,000 medicinal plant species have been recorded in the Indian Western Ghats, one of the 36 worldwide biodiversity hot spots (Gupta et al., 2022). As a country belonging to the Indian subcontinent, Bangladesh is also renowned for its herbal medicines, which feature more than 500 plant species (Rouf et al., 2021). Pakistan possesses unique biodiversity, with 1,572 genera and 5,521 species of flowering plants, of which approximately 400–600 are medicinally relevant (Bibi et al., 2015). NGE and NG have been isolated from plants in these regions, either from whole plants or specific parts such as roots and fruits. Table 1 and Table 2 summarize these plant species. The scientific names and families have been verified by Flora of China (http://www.iplant.cn/) and World Checklist of Selected Plant Families (https://wcsp.science.kew.org/).

**TABLE 1:** Isolation report of naringenin from Southeast and South Asian plants.

Serial number	Plants’ scientific name	Plants’ local name	Family	Plant part	Extract	Origin/country	Reference
1	*Salacia oblonga* Wall.	Ponkoranti	Celastraceae	Leaves	Methanol/water	India	Singh et al. (2018)
2	*Citrus reticulata* Blanco	Santra	Rutaceae	Peel	Methanol	India	Srimathi et al. (2022)
3	*Nymphaea mexicana* Zucc.	Neel kamal	Nymphaeaceae	Dried shoot	Methanol	India	Din et al. (2022)
4	*Ficus racemosa* L.	Gular	Moraceae	Fresh stem bark	Methanol/water	India	Keshari et al. (2016)
5	*Carica papaya* L.	Papaya	Caricaceae	Leaves and seeds	Extraction solvent	India	Gogna et al. (2015)
6	*Halodule pinifolia* (Miki) Hartog	Neettu korai	Cymodoceaceae	Leaves	Chloroform/methanol	India	Danaraj et al. (2020)
7	*Afzelia xylocarpa* (Kurz) Craib	Makha-hua-kham	Fabaceae	Twigs	Methanol	Thailand	Pitchuanchom et al. (2022)
8	*Wendlandia tinctoria* (Roxb.) DC.	Kara-kholi Kadam	Rubiaceae	Aerial portion	Methanol	Bangladesh	Farzana et al. (2022)
9	*Garcinia atroviridis L.*	Asam Gelugur	Clusiaceae	Dried fruits and leaves	Methanol	Indonesia	Muchtaridi et al. (2022)
10	*Bouea macrophylla* Griff.	Kundang	Anacardiaceae	Fresh leaves	Methanol	Vietnam	Nguyen et al. (2023)
11	*Hibiscus rosa sinensis* L.	Semparuthi	Malvaceae	Red flower petals	Ethanol	Vietnam	Tran Trung et al. (2020)
12	*Coix lachryma-jobi* L.	Yi-mi	Poaceae	Seeds	Ethanol	China	Chen et al. (2011)
13	*Selaginella doederleinii* Hieron.	Da-ye-cai	Selaginellaceae	Whole herbs	Ethanol/water	China	Zou et al. (2017)
14	*Craibiodendron yunnanense* W. W. Sm.	Jin-ye-zi	Ericaceae	Leaves	Ethanol	China	Wang Y. et al. (2023)
15	*Eleocharis tuberosa* Schult.	Bi-qi	Cyperaceae	Fruits	Ethanol	China	Pan et al. (2015)
16	*Iris domestica* L.	She-gan	Iridaceae	Rhizomes	Ethanol	China	Liu et al. (2022a)
17	*Malus prunifolia* (Willd.) Borkh.	Shan-zha	Rosaceae	Fresh fruits	Ethanol and ultrasounds	China	Wen et al. (2018)
18	*Smilax glaucochina* Warb.	Hei-guo-ba-qia	Smilacaceae	Rhizomes	Ethanol/water	China	Shu et al. (2018)
19	*Viburnum utile* Hemsl.	Yan-guan-jia-mi	Viburnaceae	Flowers and leaves	Ethanol	China	Long et al. (2024)
20	*Clinopodium chinense* (Benth.) Kuntze	Feng-lun-cai	Labiatae	Aerial parts	Ethanol/water	China	Zhong et al. (2012)
21	*Xanthoceras sorbifolium* Bunge	Wen-guan-guo	Sapindaceae	Husk	Ethanol	China	Li et al. (2006)
22	*Euphorbia humifusa* Willd.	Di-jin-cao	Euphorbiaceae	Whole herb	Methanol	China	Dai et al. (2024)
23	*Angelica dahurica* Hoffm.	Bai-zhi	Apiaceae	Roots	Ethanol	China	Wang M. et al. (2023)
24	Citrus aurantium L.	Zhi-qiao	Rutaceae	Raw herb	Methanol	China	Liu et al. (2018b)
25	*Morus atropurpurea* Roxb.	Guang-dong-sang	Moraceae	Seeds	Cyclohexane/ethyl acetate/n-butanol	China	Ya et al. (2006)
26	*Citrus sinensis* L.	Tian-cheng	Rutaceae	Fruits	Deuterated phosphate buffer solution	China	Lin et al. (2021)
27	*Citrus lemon* L.	Ning-meng	Rutaceae	Fruits	Deuterated phosphate buffer solution	China	Pei et al. (2022)
28	*Citrus paradisi* L.	You-zi	Rutaceae	Fruits	Deuterated phosphate buffer solution	China	Pei et al. (2022)
29	*Citrus reticulata* L.	Bu-zhi-huo	Rutaceae	Fruits	Deuterated phosphate buffer solution	China	Pei et al. (2022)
30	*Dictamnus dasycarpus* Turcz.	Bai-xian	Rutaceae	Root barks	Methanol	China	Guo et al. (2018)

**TABLE 2:** Isolation report of Naringin from Southeast and South Asian plants.

Serial number	Plants’ scientific name	Plants’ local name	Family	Plant part	Extract	Origin/country	Reference
1	*Holarrhena pubescens* Wall.	Kuthuppaalai	Apocynaceae	Leaves	Methanol	Thailand	Tuntiwachwuttikul et al. (2007)
2	*Tagetes erecta* L.	Chendu malli	Asteraceae	Petals	Ethanol/acetone/ethyl acetate/hexane	Thailand	Laovachirasuwan et al. (2024)
3	*Citrus grandis* L.	Pambalimas	Rutaceae	Peel	Methanol/water	Thailand	Caengprasath et al. (2013)
4	*Morinda citrifolia* L.	Aal	Rubiaceae	Air-dried powdered fruits	Ethanol	Malaysia	Ezzat et al. (2021)
5	Labisia pumila (Blume) Fern.-Vill	Kacip fatimah	Primulaceae	Herb	Methanol	Malaysia	Balachandran et al. (2023)
6	*Pandanus amaryllifolius* Roxb.	Biriyani Chedi, Rambha	Pandanaceae	Fresh leaves	Methanol	Malaysia	Ghasemzadeh and Jaafar (2013)
7	*Murraya koenigii* L.	Khadilimb, Karipatta	Rutaceae	Fresh leaves	Methanol	Malaysia	Ghasemzadeh et al. (2014)
8	*Hibiscus cannabinus* L.	Ambadi, Ambada	Malvaceae	Seeds	Ethanol and ultrasounds	Malaysia	Kai et al. (2015)
9	*Basella alba* L.	Poi, Pui	Basellaceae	Fresh leaves	Methanol	Malaysia	Baskaran et al. (2015)
10	*Pyrrosia longifolia* (Burm. f.) C.V.	Sulai	Polypodiaceae	Aerial parts	Methanol	Indonesia	Teruna et al. (2024)
11	*Ailanthus integrifolia* Lam.	Ai lanit	Simaroubaceae	Dried bark	CHCl_3_/methanol	Indonesia	Kosuge et al. (1994)
12	Coffea arabica L.	Kahawa, Arabic coffee	Rubiaceae	Roasting green beans	Ethanol/water	Indonesia	Hayes et al. (2023)
13	*Coffea robusta* L.	Coffee-gida	Rubiaceae	Roasting green beans	Ethanol/water	Indonesia	Hayes et al. (2023)
14	*Tadehagi triquetrum* L.	Doddotte	Fabaceae	Roots	Hydroalcoholic	India	Vedpal et al. (2020)
15	*Albizia myriophylla* Benth.	Koroi Hikaru	Fabaceae	Bark	Microwave-assisted	India	Mangang et al. (2020)
16	*Phaleria macrocarpa* (Scheff.) Boerl	Mahkota Dewa	Thymelaeaceae	Pericarp	Methanol	Indonesia	Hendra et al. (2011)
17	*Achyranthes aspera* L.	Valiya kadaladi	Amaranthaceae	Whole plants	Methanol	Bangladesh	Huq et al. (2024)
18	*Bridelia tomentosa* Blume	Khy, serai	Phyllanthaceae	Fresh leaves	Methanol	Bangladesh	Mondal et al. (2021)
19	*Neurada procumbens* L.	Chapri-booti	Neuradaceae	Mature plant	Methanol	Pakistan	Khurshid et al. (2022)
20	*Galega officinalis* L.	Citrus paradesi	Fabaceae	Fruits	Solvent evaporation	Pakistan	Mehmood et al. (2024)
21	*Morus atropurpurea* Roxb.	Guang-dong-sang	Moraceae	Seeds	Cyclohexane/ethyl acetate/n-butanol	China	Ya et al. (2006)
22	*Citrus sinensis* L.	Tian-cheng	Rutaceae	Fruits	Deuterated phosphate-buffered solution	China	Lin et al. (2021)
23	*Citrus lemon* L.	Ning-meng	Rutaceae	Fruits	Deuterated phosphate-buffered solution	China	Pei et al. (2022)
24	*Citrus paradisi* L.	You-zi	Rutaceae	Fruits	Deuterated phosphate-buffered solution	China	Pei et al. (2022)
25	*Citrus reticulata* L.	Bu-zhi-huo	Rutaceae	Fruits	Deuterated phosphate-buffered solution	China	Pei et al. (2022)
26	*Davallia trichomanoides* Blume	Gu-sui-bu	Davalliaceae	Dried rhizome	Methanol	China	Dong et al. (2023)
27	*Selliguea hastata* (Thunb.) H.	Jin-ji-jiao	Polypodiaceae	Dried rhizome	Ethanol	China	Duan et al. (2012)
28	*Euphorbia humifusa* Willd.	Di-jin	Euphorbiaceae	Whole herb	Methanol	China	Dai et al. (2024)
29	*Angelica dahurica* Fisch.	Bai-zhi	Apiaceae	Roots	Ethanol	China	Wang M. et al. (2023)
30	*Litchi chinensis* Sonn.	Li-zhi	Sapindaceae	Seeds	Ethanol	China	Liu et al. (2019)

## 3 Methods

This review was conducted by searching three databases (PubMed, Web of Science, and Scopus) up to January 2025, using keywords such as “naringenin,” “naringin,” “ischemia reperfusion,” “reperfusion injury,” “stroke,” and “infarction.” The inclusion criteria were as follows: 1) IRI models, including both *in vitro* and *in vivo* studies, regardless of organ, gender, age, species, or language; 2) the presence of a control group; and 3) at least one experimental group using NGE or NG as an intervention. The exclusion criteria were as follows: 1) conference papers, reviews, comments, case reports, posters, and editorials; 2) incomplete data; and 3) studies on other compounds and diseases. As shown in File 1, the complete electronic search strategy was implemented across the three databases.

## 4 Chemical properties and pharmacokinetics of NGE and NG

Structurally, NGE (4′,5,7-trihydroxyflavanone; molecular weight: 272.256 g/mol) consists of two benzene rings (A and B) connected by a chromane ring (C ring). There are three hydroxyl groups on the ring: one at position 4′ on the B ring and two at positions 5 and 7 on the A ring (Joshi et al., 2018; Kaźmierczak et al., 2025). There is one glycosylated derivative of NGE called NG (4′,5,7-trihydroxyflavanone-7-rhamnoglucoside; molecular weight: 580.5 g/mol) (Shilpa et al., 2023). Compared to NGE, NG has a rhamnoside group attached to C7 in the A ring. As a result of their hydroxyl groups (7-OH, 4′-OH, and 5-OH groups), which are responsible for free radical scavenging and metal ion chelating properties, NGE and NG are potent antioxidant agents (Panche et al., 2016; Rani et al., 2016).

As soon as NGE and NG reach the cells, they undergo phase I metabolism by cytochrome P450 monooxygenases (oxidation or demethylation), followed by phase II metabolism by intestinal cells or liver cells (glucuronidation, sulfation, or methylation) (Kawaguchi et al., 2011; Yang X. et al., 2022; Yang Y. et al., 2022). Microbes in the gut further metabolize metabolites and unabsorbed flavonoids into catabolites of phenolic and aromatic rings, boosting their bioavailability (Kay et al., 2017). There are two common ways to excrete NGE and NG: through bile and urine (Joshi et al., 2018). Bile excretes metabolites into the enterohepatic circulation, prolonging their elimination half-life (Ma et al., 2006). It has been demonstrated that NGE and NG are extensively distributed in certain rat tissues, such as the gut, liver, kidneys, lungs, and trachea (Zeng et al., 2019). There is evidence that NGE and NG can cross the blood–brain barrier (BBB), possibly through passive diffusion or active transport (Lai et al., 2025). In addition, new evidence suggests that gender and age are associated with pharmacokinetics, which in turn affects bioactivity *in vivo* (Yang Y. et al., 2022). Due to poor oral absorption, first-pass metabolism, and metabolite elimination, NGE and NG only have 5%–15% oral bioavailability, which limits their clinical potential (Flores-Peña et al., 2025; Joshi et al., 2018; Lavrador et al., 2018). Various strategies have been examined to improve the bioavailability of NGE and NG, including polymeric nanoparticles, hydrogel nanocarriers, lipid nanoparticles, magnetic nanoparticles, and nanoemulsions (Flores-Peña et al., 2025). There is no doubt that these novel approaches have the potential to enhance the therapeutic effects of NGE and NG. However, their advantages and limitations must be carefully evaluated prior to clinical application.

## 5 Comparison of NGE and NG

Apart from their chemical structures, NGE and NG are different in many aspects. In some studies, NGE and NG pharmacokinetics have been compared. A mean concentration–time profile in plasma was calculated after oral administration of NGE and NG to rabbits, and NG and its conjugates reached maximal concentrations after nearly 90 min, whereas NGE peaked after 10 min (Hsiu et al., 2002). A study on RAW264.7 cells showed that NGE had a weaker anti-inflammatory effect than NG in response to lipopolysaccharide-stimulated inflammation. NGE reduced inflammation by inhibiting nuclear factor-κB (NF-κB) and p38 phosphorylation, and NG reduced inflammation by inhibiting NF-κB and mitogen-activated protein kinases (MAPK) (Cho and Shaw, 2024). Additionally, researchers have found that NGE scavenges hydroxyl and superoxide radicals more effectively than NG (Cavia-Saiz et al., 2010). As a result of its sugar group partially blocking access to active hydroxyl groups, NG has lower effectiveness at neutralizing free radicals (Li P. et al., 2020). Currently, only limited research compares NGE and NG effects. Hence, the varied bioactivities between NGE and NG necessitate further research.

## 6 Pharmacokinetic interactions and clinical safety considerations

It has been demonstrated that NGE and NG are the primary culprits in grapefruit-related food–drug interactions (Memariani et al., 2021). Cytochrome P450 enzymes participate in drug metabolism and detoxify carcinogens (Burkina et al., 2016). Research shows that NGE and NG inhibit cytochrome P450 enzymes, which can increase drug blood concentrations when co-administered with other drugs (Fuhr and Kummert, 1995). Further research has found that both NGE and NG inhibit the cytochrome P450 enzymes 1A2 and 3A4, which belong to the subfamily of cytochrome P450 enzymes (Burkina et al., 2016; Fuhr et al., 1993). Carboxylesterase 1 is a major esterase present in the liver, and it hydrolyzes a wide variety of therapeutic agents and toxins. In one study, NGE showed the highest inhibition potential for carboxylesterase 1 (Qian and Markowitz, 2020). In addition, NGE inhibited P-glycoprotein activity, another efflux transporter (Romiti et al., 2004). In IRI patients, some drugs (including antidepressants, anti-infectives, calcium antagonists, and statins) can interact with grapefruit juice (Kane and Lipsky, 2000). Due to these food–drug interactions, NGE and NG should be used cautiously when co-administered with certain drugs.

Currently, insufficient clinical studies have been conducted because NGE and NG are poorly soluble in water and have low bioavailability. One study found that among healthy adults, ingestion of 150–900 mg of NGE was safe (Rebello et al., 2020). Commercial grapefruit juices contain NG concentrations ranging from 50 to 1,200 mg/L (Ho et al., 2000), and some dietary supplements recommend doses of 200–1,000 mg/day of NG (Jung et al., 2003). For a more comprehensive understanding of safety and efficacy in humans, further clinical studies are urgently needed.

## 7 Protective effect of NGE and NG against IRI

Experimental studies have found that NGE and NG improve IRI in the myocardium, brain, intestines, kidneys, and other organs (Table 3; Table 4). Through this review, we found that NGE and NG alleviated IRI through multiple pathways involving the inhibition of oxidative stress, inflammation, apoptosis, endoplasmic reticulum (ER) stress, ferroptosis, and pyroptosis and the regulation of autophagy.

**TABLE 3:** Protective effect of NGE against organ IRI.

Type of disease	Experimental model	Dose/route	Key quantitative outcome	Primary proposed mechanism	Reference
Myocardial IRI	Male SD rats (I/R: 30 min/4, 6 h)	50 mg/kg/days, i.g., 5 days	↑LVSP, +dP/dtmax, −dP/dtmax ↓Infarct size	Anti-apoptotic Anti-oxidative stress Anti-ER stress Activating cGMP-PKGIα signaling	Yu et al. (2019b)
	H9C2 cells (H/R: 1 h/4 h)	80 μM, 6 h	↑Cell viability		
	Male SD diabetic rats (I/R: 30 min/2 h)	25 and 50 mg/kg/days, p.o., 30 days	↑LVSP, ±dP/dtmax ↓Infarct size, CK-MB, LVEDP	Anti-oxidative stress Upregulating the miR-126-PI3K/AKT axis	Li et al. (2021b)
	SD rats (I/R: 30 min/4 h)	10 and 50 mg/kg/days, i.g., 7 days	↓Myocardial infarct size	Anti-ferroptosis Regulating the Nrf2/system xc-/GPX4 axis	Xu et al. (2021)
	H9C2 cells (H/R: 6 h/12 h)	20, 40, and 80 μM, 24 h	↑Cell viability		
	H9C2 cells (H/R: 6 h/12 h)	40, 80, and 160 μM	↑Cell viability	Anti-ER stress Anti-apoptotic	Tang et al. (2017)
	Male SD rats (I/R: 30 min/4, 6 h)	50 mg/kg/days, 7 days	↑LVSP, +dP/dtmax, −dP/dtmax ↓Infarct size	Anti-oxidative stress Anti-apoptotic Activating AMPK–SIRT3 signaling	Yu et al. (2019a)
	H9C2 cells (H/R: 1 h/4 h)	80 μM, 6 h	↑Cell viability		
	Male SD rats (I/R: 30 min/2 h)	50 mg/kg/days, i.g., 7 days	↓Myocardial pathological damage	Regulating the miR-24-3p/Cdip1 axis	Jin et al. (2024b)
	H9C2 cells (H/R: 6 h/24 h)	80 μM, 1 h	↑Cell viability		
	Male Wistar rats (I/R: 30 min/2 h)	100 mg/kg, i.p.	↑LVDP, dP/dt ↓Injured areas	Activating mitochondrial BK channels	Testai et al. (2013b)
	Male SD rats (I/R: 30 min/1–2 h)	1.25, 2.5, 5, 10, 20, and 40 μM	↑LVDP, +LVdP/dtmax, –LVdP/dtmax ↓LVEDP, infarct area	Anti-oxidative stress	Meng et al. (2016)
	One-year-old male Wistar rats (I/R: 30 min/2 h)	100 mg/kg, i.p.	↓Ai/ALV, calcium up-take, Tl influx into the matrix	Activating the mitoBK channel;	Testai et al. (2017)
	Senescent H9C2 cells (H/R: 16 h/2 h)	4–40 μM, 16 h	↑Cell viability		
	SD rats (I/R: 45 min/4 h)	2.5, 5, and 10 mg/kg;	↓CK-MB	Anti-oxidative stress Anti-apoptotic	Ren et al. (2022)
	H9C2 cells (H/R: 6 h/16 h)	1.25, 2.5, 5, 10, 20, 40 μM, 12 h	↑Cell viability		
	Male Wistar rats (I/R: 30 min/2 h)	100 mg/kg, i.p.	↑Rate pressure product, time required to reach half-maximal ischemic contracture ↓Ischemic area	​	Testai et al. (2013a)
	Male SD rats (I/R: 30 min/2 h)	100 mg/kg, i.p., 7 days;	↓cTnI, myocardial pathological damage, infarction area	Anti- ER stress Anti-apoptotic Activating the PI3K/AKT pathway	Lan et al. (2021)
Cerebral IRI	Male Wistar rats (I/R: 2 h/24 h)	50 and 100 mg/kg/days, p.o., 30 days	↑Cognitive function	Anti-oxidative stress; anti-inflammatory Upregulating BDNF/TrkB signaling	Zhu and Gao (2024)
	HT22 cells (OGD/R:6 h/24 h)	80 μM, 24 h	↑Cell viability	Anti-oxidative stress Anti-apoptosis Anti-inflammatory Activating SIRT1/FOXO1 signaling	Zhao et al. (2023)
	Male Wistar rats (I/R: 1 h/23 h)	50 mg/kg/days, p.o., 21 day	↑Motor coordination skill, grip strength, neurological deficits, tapes removal test ↓Infarct volume, neuronal damage	Suppressing NF-κB mediated neuroinflammation	Raza et al., 2013
	Male Wistar rats (I/R: 1 h/1 h)	50 and 100 mg/kg/days, i.p., 14 days	↓AQP4;	Anti-DNA damage Anti-inflammatory	Somuncu, et al., 2021
	Male SD rats (I/R: 30 min/5.5 h)	0.1, 1, and 10 mg/kg, iv.	↓Infarct volume;	Anti-oxidative stress	Saleh et al. (2017)
	Mixed neuronal (OGD/R: 24 h/24 h)	0.25–1,250 μM, 24 h	​		
	Male SD rats (I/R: 2 h)	80 μM, i.p.	↓mNSS score, Wet-Dry weight ratio;	Anti-apoptotic Anti-oxidative stress	Wang et al. (2017)
	Cortical neurons (OGD/R: 2 h/24 h)	20, 40, and 80 μM, 48 h	↑Cell viability;		
	Male C57BL/6 mice (I/R: 1 h/7 d)	1, 5, 10, and 20 mg/kg/day, i.p., 6 days	↑Neurological deficits ↓Infarct volumes	Anti-inflammatory Activating the GSK-3β/β-catenin pathway;	Yang et al. (2024)
Intestinal IRI	Male C57BL/6J mice (I/R: 45 min/30 min)	50 and 100 mg/kg/days, i.g., 7 days	↓Chiu’s score	Anti-oxidative stress; anti-ferroptosis	Hou et al. (2024)
	IEC-6 cells (OGD/R: 3 h/1 h)	50, 75, and 100 μM, 12 h	↑Cell viability		
Renal IRI	Male C57Bl/6 mice (I/R: 30 min/24 h)	50 mg/kg/days, i.g., 3 days	↓Cr, BUN, kidney injury scores	Anti-ER stress Anti-pyroptosis Anti-apoptosis Activating Nrf2/HO-1 signaling	Zhang et al. (2022)
	HK-2 cells (H/R: 12 h/4 h)	5 mM for 24 h	​		
	Male Wistar rats (I/R: 45 min/24 h)	50 mg/kg/days, i.p., 7 days	↓Renal tubular injury score, Cr	Anti-inflammatory Inhibiting the NF-κB pathway	Dai et al. (2021)
Retinal IRI	Male Wistar rats (I/R: 1 h/24 h)	20 mg/kg, i.p.	↑Retinal thickness ↓Retinal damage	Anti-apoptosis	Kara et al. (2014)
	Male C57BL/6 mice (I/R: 1 h/7 d)	100 and 300 mg/kg, i.g., 7 days	↑Number of cells in ganglion cell layer, ganglion cell layer thickness ↓Ocular hypertension	Inhibiting neuroinflammation Regulating CD38/SIRT1 signaling	Zeng et al. (2024)
​	Female Long–Evans rats (I/R: 30 min/240 min)	10 mg/kg, i.p.;	↑b-wave of electroretinogram;	​	Chiou and Xu, 2004

Abbreviations: AKT, protein kinase B; AMPK, adenosine monophosphate-activated protein kinase; AQP4, aquaporin-4; BDNF, brain-derived neurotrophic factor; BUN, blood urea nitrogen; CK, creatine kinase; Cr, creatinine; cGMP, cyclic guanosine monophosphate; ER, endoplasmic reticulum; FOXO1,: forkhead box O1; GPX, glutathione peroxidase; GSK, glycogen synthase kinase; H/R, hypoxia–reoxygenation; HO-1, heme oxygenase (decycling) 1; i. g., intragastric; i. p., intraperitoneal; i. v., intravenous; I/R, ischemia/reperfusion; LVEDP, left ventricular end-diastolic pressure; LVSP, left ventricular systolic pressure; NF-κB, nuclear factor-kappa B; Nrf2, nuclear factor E2-related factor 2; OGD/R, oxygen and glucose deprivation/reperfusion; p.o., oral administration; PI3K, phosphatidylinositol 3-kinase; PKGIα, protein kinase GIα; SIRT, silent information regulator; TrkB, tyrosine receptor kinase B.

**TABLE 4:** Protective effect of NG against organ IRI.

Type of disease	Experimental model	Dose/route	Key quantitative outcome	Primary proposed mechanism	Reference
Myocardial IRI	AC16 cells (OGD/R: 6 h/6 h)	200 μM	↑ Cell viability	Anti-apoptotic, anti-inflammatory, regulating miR-126/GSK-3β/β-catenin signaling	Guo et al. (2022)
	Male SD rat (I/R: 30 min/1–7 days)	50 mg/kg	↓ Myocardia damage		
	Male wistar rats (I/R: 40 min/45 min)	40 and 100 mg/kg/day, i.g., 8 weeks	↑ LVDP, dp/dtmax ↓Myocardial hypertrophy, CK-MB	Anti-oxidative stress, anti-inflammatory	Shackebaei et al. (2024)
	Male SD rat (I/R: 30 min/3 h)	25, 50, and 100 mg/kg/days, i.p., 7 days	↑ Fractional shortening ↓ CK-MB, cTnI, damage score, infarct size	Anti-apoptotic, anti-inflammatory Anti-oxidative stress Anti-autophagy Activating PI3K/AKT signaling	Li et al. (2021a)
	Male Wistar rats (I/R: 30 min/1 h)	25 mg/kg/days, 7 days	↑ LVDP, +dP/dt, −dP/dt; ↓ Arrhythmia episodes, myocardial infarct area	Anti-apoptotic Anti-oxidative stress	Araujo et al. (2023)
	Male Wistar rats (I/R: 45 min/1 h)	20, 40, and 80 mg/kg/days, p.o., 14 days	↑ ±LVdP/dt max ↓ LVEDP, CK-MB	Anti-inflammatory Anti-oxidative stress Regulating Hsp27, Hsp70, p-eNOS/p-Akt/p-ERK signaling	Rani et al. (2013)
	Male SD rats (I/R: 30 min/4 h)	5 mg/kg, i.p., 5 days	↓Myocardial infarction size, CK-MB, cTnl	Anti-apoptosis, anti-inflammatory Anti-oxidative stress Activating SIRT1 signaling	Liu et al. (2022a)
Cerebral IRI	Male SD rats (I/R: 30 min/2 h)	10, 50, and 100 mg/kg, i.p.	↓ Myocardial infarction area, myocardial pathological damage, CK	Anti-pyroptosis	Wang T. T. et al. (2021)
	Male SD rats (I/R: 30 min/2 h)	10, 20, and 40 mg/kg/days, i.g., 7 days	↓ Apoptotic cells	Anti- ER stress Anti-apoptotic	Liu et al. (2018a)
	Male Wistar rats (I/R: 30 min/7.14 days)	100 mg/kg/days, i.g., 14 days	↓ Neurologic score, rotarod test values	Increasing neurogenesis	Yilmaz et al. (2024)
	Male SD rats (I/R: 2 h/24 h)	5 mg/kg/days, i.p., 7 days	↓ Brain water content, cerebral infarction volume, neurological deficit score	Anti-apoptosis, anti-inflammatory Activating the PI3K/AKT pathway	Yang et al. (2020)
	Primary nerve cells (OGD: 72 h)	6, 12, and 25 μg/mL, 48 h	↑ Cell viability		
	SH-SY5Y cells (OGD/R: 10 h/14 h)	12.5, 25, 50, 100, 150, and 200 μM, 24 h	↑ Cell viability	Attenuating mitophagy	Feng et al. (2018)
	Male SD rats (I/R: 2 h/22 h	120 mg/kg, i.v.	↓ Neurological deficit score, pathomorphological injury, infarct size		
	PC12 cells (OGD/R: 2 h/12 h)	0, 1, 5, 10, and 50 μM	↑ Cell viability	Anti-apoptosis, Targeting NFKB1; Modulating HIF-1α/AKT/mTOR signaling	Cao et al. (2021)
	Male Wistar rats (I/R: 30 min/24 h)	50 and 100 mg/kg, i.p., 7 days	↑ Hanging latency time, locomotor activity ↓ Neurological score, pathomorphological injury	Anti-oxidative stress Anti-free radical scavenging property	Gaur et al. (2009)
	Females and males, SD rats (I/R: 2 h/24 h)	25, 50, and 100 mg/kg/days, i.g., 7 days	↓ Zea-Longa score, infarct size, brain water content	Anti-oxidative stress, anti-inflammatory, anti-ER stress	Wang L. et al. (2021)
Intestinal IRI	Male Wistar rats (I/R: 2 h/2 h)	50 mg/kg, i.p.	↓ Chiu’s score	Anti-oxidative stress	Bakar et al. (2019)
	Male Wistar rats (I/R: 2 h/2 h)	50 mg/kg, i.p.	​	Anti-oxidative stress	Cerkezkayabekir et al. (2017)
	Male SD rats (I/R: 1 h/2 h)	50 and 100 mg/kg/days, i.g., 3 days	↓ Chiu’s score	Anti-oxidative stress Anti-apoptosis, anti-inflammatory Deactivating cGAS–STING signaling	Gu et al. (2023)
	IEC-6 cells (H/R: 12 h/4 h)	16, 32, 64, 128, and 256 μM, 12 h	↑Cell viability		
	Male SD rats (I/R: 1 h/1 h)	80 mg/kg, i.p.	↓ Chiu’s score	​	Isik et al. (2015)
Renal IRI	Male Wistar rats (I/R: 45 min/72 h)	50 and 100 mg/kg/day, i.p., 3 days	↓ HE staining score	Anti-inflammation Anti-apoptosis	Danis et al. (2024)
	Male SD rats (I/R: 45 min/4 h)	100 mg/kg/days, i.p., 7 days	​	Anti-oxidative stress Activating Nrf2 expression	Amini et al. (2019a)
	Male SD rats (I/R: 45 min/24 h)	400 mg/kg, p.o.	↑ Creatinine clearance ↓ Histopathological injury, Cr, BUN	Anti-oxidative stress	Singh and Chopra (2004)
	Male SD rats (I/R: 45 min/4 h)	100 mg/kg/days, i.p., 7 days	↑ Renal blood flow, creatinine clearance, fractional excretion of sodium ↓ Histopathology scoring, BUN, Cr	Anti-oxidative stress Anti-apoptosis	Amini et al. (2019b)
​	Male SD rats (I/R: 45 min/4 h)	100 mg/kg/days, i.p., 7 days	↑ Nucleus tractus solitarius electrical activity, baroreceptor sensitivity	​	Amini et al. (2021)
​	Male SD rats (I/R: 45 min/4 h)	100 mg/kg/days, i.p., 3 days	​	Anti-oxidative stress Preserving the kidney mitochondria	Amini and Goudarzi,2022
Hindlimb IRI	Male SD rats (I/R: 2 h/2 h)	400 mg/kg, p.o., three times with an 8 h interval	​	Anti-oxidative stress	Gürsul et al., 2016
Testicular IRI	Male Wistar rats (I/R: 4 h/4 h)	5 and 10 mg/kg, i.p.	↓ Histological injury	Anti-oxidative stress	Akondi et al. (2011)
Spinal cord IRI	Female SD rats (I/R: 30 min/1–7 days)	50 and 100 mg/kg, i.p., 7 days	↑ Locomotor function, BBB score ↓ Spinal edema	Anti-inflammatory Anti-oxidative stress	Cui et al. (2017)
Liver IRI	Male SD rats (I/R: 70 min/2 h)	80 mg/kg, i.p.	​	Anti-oxidative stress	Gursul et al., 2017

Abbreviations: AKT, protein kinase B; BBB, Basso, Beattie, and Bresnahan; BUN, blood urea nitrogen; cGAS, cyclic guanosine monophosphate–adenosine monophosphate synthase; CK, creatine kinase; Cr, creatinine; ER, endoplasmic reticulum; GSK, glycogen synthase kinase; HIF-1α, hypoxia-inducible factor-1α; i. g., intragastric; i. p., intraperitoneal; I/R, ischemia/reperfusion; LVDP, left ventricular diastolic pressure; mTOR, mammalian target of rapamycin; Nrf2, nuclear factor E2-related factor 2; OGD/R, oxygen and glucose deprivation/reperfusion; p. o., oral administration; PI3K, phosphatidylinositol 3-kinase; SIRT, silent information regulator; STING, stimulator of interferon genes.

### 7.1 Inhibition of oxidative stress

Several studies have concluded that oxidative stress leads to IRI (Yi et al., 2023). During oxidative stress, cells are exposed to pathogenic stimuli, which produce reactive oxygen species (ROS) and reactive nitrogen oxides (Zhang et al., 2025). Superoxide dismutase (SOD), catalase (CAT), glutathione (GSH), and glutathione peroxidase (GPX) are endogenous antioxidant enzymes that eliminate free radicals (Yuan et al., 2021). The imbalance between antioxidants and oxidation results in cell death, which is a major cause of IRI (Ramachandra et al., 2020). Several *in vivo* and *in vitro* studies have shown that NGE and NG protect against IRI by attenuating oxidative stress. In the IRI models of the myocardium, brain, intestines, kidneys, hindlimb, testicles, spinal cord, and liver, NGE and NG increased SOD, GPX, GSH, and CAT levels and reduced ROS and malondialdehyde (MDA) levels (Akondi et al., 2011; Amini and Goudarzi, 2022; Amini et al., 2019a; b; Araujo et al., 2023; Bakar et al., 2019; Cao et al., 2021; Cui et al., 2017; Gaur et al., 2009; Gu et al., 2023; Gürsul et al., 2016; Gursul et al., 2017; Hou et al., 2024; Isik et al., 2015; Jin X. et al., 2024; Li F. et al., 2021; Li S. H. et al., 2021; Liu Y. et al., 2022; Meng et al., 2016; Rani et al., 2013; Raza et al., 2013; Ren et al., 2022; Saleh et al., 2017; Shackebaei et al., 2024; Singh and Chopra, 2004; Wang et al., 2017; Wang T. T. et al., 2021; Wang T. T. et al., 2021; Xu et al., 2021; Yu et al., 2019a; Yu et al., 2019b; Zhao et al., 2023; Zhu and Gao, 2024). 8-hydroxydeoxyguanosine (8-OHdG) is a product of oxidative metabolism (Somuncu et al., 2021). In diabetic myocardial IRI rats and cerebral IRI rats, NGE decreased 8-OHdG levels (Li S. H. et al., 2021; Somuncu et al., 2021). Furthermore, IRI increases the activity of inducible nitric oxide synthase (iNOS) and neuronal nitric oxide synthase (nNOS), thereby promoting nitric oxide (NO) formation (Yuan et al., 2021). When NO combines with the superoxide anion to form peroxynitrite, it induces oxidative stress (Wu et al., 2010). NG possessed a strong peroxynitrite (ONOO^−^) scavenging capability (Feng et al., 2018). In cerebral and intestinal IRI models, NG inhibited iNOS and nNOS expressions and prevented NO release (Cerkezkayabekir et al., 2017; Feng et al., 2018; Raza et al., 2013). Researchers have found that arginase inhibits oxidative stress by regulating NO production in IRI. In intestinal IRI, arginase activity was significantly elevated, whereas NG alleviated oxidative stress by reducing arginase activity (Cerkezkayabekir et al., 2017).

### 7.2 Inhibition of inflammation

Inflammation is considered a crucial factor in pathophysiological variations following IRI (Mo et al., 2020). In IRI, neutrophils are recruited and release pro-inflammatory cytokines, including interleukin (IL)-1β, IL-6, and tumor necrosis factor (TNF)-α, exacerbating cellular damage (Hou et al., 2023). Several studies have reported that NGE and NG have anti-inflammatory effects on IRI. In IRI models of the myocardium, brain, intestines, kidneys, and spinal cord, NGE and NG reduced levels of pro-inflammatory cytokines (IL-1β, IL-6, and TNF-α) (Cui et al., 2017; Dai et al., 2021; Danis et al., 2024; Gu et al., 2023; Guo et al., 2022; Li F. et al., 2021; Liu Y. et al., 2022; Rani et al., 2013; Raza et al., 2013; Shackebaei et al., 2024; Wang L. et al., 2021; Wang T. T. et al., 2021; Yang et al., 2020; Yang et al., 2024; Zhang et al., 2022; Zhao et al., 2023; Zhu and Gao, 2024). Meanwhile, NGE and NG remarkably increased IL-10 levels in middle cerebral artery occlusion and reperfusion (MCAO/R)-induced cerebral IRI rats and in oxygen and glucose deprivation/reperfusion (OGD/R)-induced HT22 cells (Wang l. et al., 2021; Zhao et al., 2023). In intestinal IRI-induced rats and hypoxia–reoxygenation (H/R)-induced IEC-6 cells, NG reduced interferon (IFN)-β levels (Gu et al., 2023). Myeloperoxidase (MPO) is an enzyme secreted by neutrophils and macrophages during inflammation, and it is highly expressed during IRI (Netala et al., 2025). It has been shown that NGE and NG reduced MPO levels in the IRI of the myocardium, brain, intestines, and spinal cord (Cui et al., 2017; Gu et al., 2023; Raza et al., 2013; Xu et al., 2021). As a key rate-limiting enzyme, cyclooxygenase (COX) plays a critical role in the biosynthesis of prostanoids, which activate complex inflammatory cascades (Jiang and Yu, 2021). A study of MCAO/R-induced cerebral IRI rats found that NGE treatment decreased COX-2 expression (Raza et al., 2013).

### 7.3 Inhibition of apoptosis

Apoptosis is a programmed cell death process activated by IRI (Sun et al., 2025). According to the studies, apoptosis occurs in almost all IRI pathological processes (Zhai et al., 2024). Following IRI, expression of B-cell lymphoma-2 (Bcl-2) was significantly downregulated, whereas Bcl-2-associated X (Bax) and cleaved caspase-3 were significantly elevated (Yu et al., 2019a). Numerous experiments have shown that NGE and NG protect IRI through anti-apoptotic mechanisms. In IRI models of the myocardium, brain, intestines, and kidneys, NGE and NG reduced apoptosis index, cleaved caspase-3, and Bax levels and significantly increased Bcl-2 expression (Amini et al., 2019b; Araujo et al., 2023; Cao et al., 2021; Danis et al., 2024; Gu et al., 2023; Guo et al., 2022; Kara et al., 2014; Lan et al., 2021; Li F. et al., 2021; Liu Y. et al., 2022; Rani et al., 2013; Ren et al., 2022; Tang et al., 2017; Wang et al., 2017; Yang et al., 2020; Yu et al., 2019a; Yu et al., 2019b; Zhang et al., 2022; Zhao et al., 2023). Caspase-9, an essential caspase that initiates apoptosis *via* the mitochondrial pathway, is activated during the apoptotic process (Würstle et al., 2012). In a study of OGD/R-induced cortical neurons, NGE inhibited caspase-9 (Wang et al., 2017).

### 7.4 Inhibition of ER stress

The presence of ER stress also contributes to IRI (Feng et al., 2019). During IRI, free radicals and hypoxia exposure disrupt ER homeostasis, leading to ER stress (Feng et al., 2019). ER stress is triggered by accumulating misfolded and unfolded proteins, which are marked by glucose-regulated protein 78 (GRP78), unfolded protein response (UPR), and CCAAT/enhancer binding protein homologous protein (CHOP) (Yu et al., 2019b; Zhang J. et al., 2024). Meanwhile, ER stress is mediated by three ER stress sensors: inositol-requiring enzyme 1α (IRE1α), activating transcription factor 6 (ATF6), and protein kinase R-like ER kinase (PERK) (Jin H. et al., 2024). Caspase-12 is found in the ER cytoplasm and mediates the death of cells exposed to ER stress (Momoi, 2004). According to studies, NGE and NG significantly inhibited ER stress, as evidenced by a reduction in CHOP, GRP78, and cleaved caspase-12 in myocardial IRI, cerebral IRI, and renal IRI (Lan et al., 2021; Liu D. et al., 2018; Tang et al., 2017; Wang L. et al., 2021; Yu et al., 2019b; Zhang et al., 2022). In myocardial IRI rats and H/R-induced H9C2 cells, NGE also decreased ATF6 (Tang et al., 2017; Yu et al., 2019b). NGE treatment inhibited ER stress *via* activation of cyclic guanosine monophosphate-dependent protein kinase Iα signaling (Yu et al., 2019b). Meanwhile, in MCAO/R-induced cerebral IRI rats, NG also improved ER stress by decreasing ATF6 (Wang L/et al., 2021).

### 7.5 Inhibition of ferroptosis

Ferroptosis is a significant driver of IRI and organ failure, especially in the later phases of reperfusion (Cai et al., 2023). In contrast to other forms of cell death, ferroptosis causes mitochondrial dysfunction, abnormal iron metabolism, and an imbalance in the GPX4/GSH axis (Li J. et al., 2020). In several studies, NG and NGE inhibited ferroptosis, protecting against IRI. In myocardial IRI rats, NGE significantly reduced Fe^2+^ and total iron levels, while ferritin heavy chain 1, solute carrier family 7 member 11 (SLC7A11), and GPX4 levels were significantly increased (Xu et al., 2021). In a study of intestinal IRI, NGE decreased long-chain acyl-CoA synthetase 4 and iron levels while increasing GPX4 and SLC7A11 levels (Hou et al., 2024). In intestinal IRI, NGE inhibited ferroptosis by activating Yes-associated protein, which regulated signal transducer and activator of transcription 3 phosphorylation (Hou et al., 2024).

### 7.6 Regulation of autophagy

Among metabolic pathways, autophagy plays a crucial role in cell survival (Ding et al., 2024). Autophagy involves the engulfment of organelles and metabolic waste into the autophagosome, which then fuses with the lysosome to eliminate them (Debnath et al., 2023). In IRI, autophagy plays a double-edged role (Ding et al., 2024). When autophagy is activated during ischemic conditions, metabolic waste is removed and protects cells, whereas excessive autophagy upon reperfusion results in the depletion of intracellular components and eventual cell death (Mokhtari and Badalzadeh, 2022). Autophagy can be assessed by several autophagy-related proteins, including light chain (LC) 3B, Beclin 1, and P62 (Gao et al., 2022). A study of myocardial IRI found that NG enhanced autophagy levels by increasing autophagosome formation, autophagy flux, Beclin 1, and LC3B-II/LC3B-I while reducing P62 (Li F. et al., 2021). In cerebral IRI, researchers found that NG lowered the ratio of LC3B-II/LC3B-I in mitochondrial fractions and inhibited Parkin translocation into mitochondria (Feng et al., 2018).

### 7.7 Inhibition of pyroptosis

Unlike necrosis and apoptosis, pyroptosis is an inflammatory form of cell death that causes the formation of plasma membrane pores, cell swelling, osmotic lysis, and the release of inflammatory factors such as IL-1β and IL-18 (Du et al., 2025). Pyroptosis can be induced by caspase-1, 4, 5, or 11 and mediated by gasdermin D (GSDMD) (Zhang S. et al., 2024). Apoptosis-associated speck-like protein (ASC) and procaspase-1 are recruited by Nod-like receptor protein 3 (NLRP3) to form inflammasomes, which initiate pyroptosis. In turn, this triggers GSDMD, which leads to cell death, also known as the classical pathway to pyroptosis (Liu W. et al., 2022). Pyroptosis is crucial to IRI progression. Many experiments have demonstrated that NGE and NG inhibit pyroptosis in IRI. In myocardial IRI rats, NG inhibited pyroptosis by decreasing ASC, caspase-1, NLRP3, and GSDMD levels (Wang L. et al., 2021). It was also found that NGE significantly inhibited the pyroptosis-related markers, including NLRP3, ASC, and caspase-1, in renal IRI (Zhang et al., 2022).

### 7.8 Other mechanisms

There is evidence that ischemia triggers neurogenesis in the usual neurogenic niches, namely, the subventricular zone of the lateral ventricle and the subgranular zone of the dentate gyrus (Jhelum et al., 2022). It was found that NG significantly increased neurogenesis in cerebral IRI rats by restoring the mature neuron marker (NeuN) and immature neuron marker (DCX) (Yilmaz et al., 2024). In terms of adult neurogenesis and stroke-induced neurogenesis, brain-derived neurotrophic factor (BDNF) is the most extensively researched neurotrophic factor (Leal et al., 2017). An increase in BDNF levels enhanced learning, memory, and function in animals induced by cerebral IRI (Ni et al., 2020). A study found that NG increased BDNF levels in the cerebral IRI, contributing to neurogenesis (Yilmaz et al., 2024).

As a specific barrier between the blood and the brain, the BBB maintains brain stability by regulating the transport of beneficial and harmful substances between the blood and the brain (Hu et al., 2024). After cerebral IRI, BBB integrity is compromised, resulting in increased paracellular permeability and allowing the passage of toxins, inflammatory factors, and immune cells into the brain, ultimately leading to brain edema and death (Liao et al., 2020). Strategies targeting BBB integrity may be valuable for preventing disability and enhancing recovery after cerebral IRI (Hou et al., 2023). Tight junction (TJ) proteins are critical for BBB integrity. It has been found that ischemic injuries result in reduced levels of TJ proteins, including zonula occludens 1 (ZO-1) and claudin-5 (Hu et al., 2024). In MCAO/R-induced cerebral IRI rats, NGE increased TJ expression (occludin, claudin-5, and ZO-1) and prevented Evans blue dye leakage (Yang et al., 2024). It has been found that GSK-3β/β-catenin signaling is required for NGE-alleviated BBB in cerebral IRI (Yang et al., 2024).

## 8 Signaling pathways and target proteins of NGE and NG in protection against IRI

### 8.1 NF-κB signaling pathway

As a classical signaling pathway, the NF-κB pathway is involved in inflammation, oxidative stress, and cell death (Gasparini and Feldmann, 2012). At rest, NF-κB exists in the cell as an inactive dimer bound to the inhibitor protein (IκB) (Karin, 1999). After IRI, cells are stimulated, and IκB proteins are degraded through phosphorylation, triggering NF-κB activation (Li et al., 2022). Activated NF-κB translocates into the nucleus and exerts its transcriptional function, enhancing the production of inflammatory factors and ultimately aggravating IRI (van Delft et al., 2015).

**FIGURE 1:**
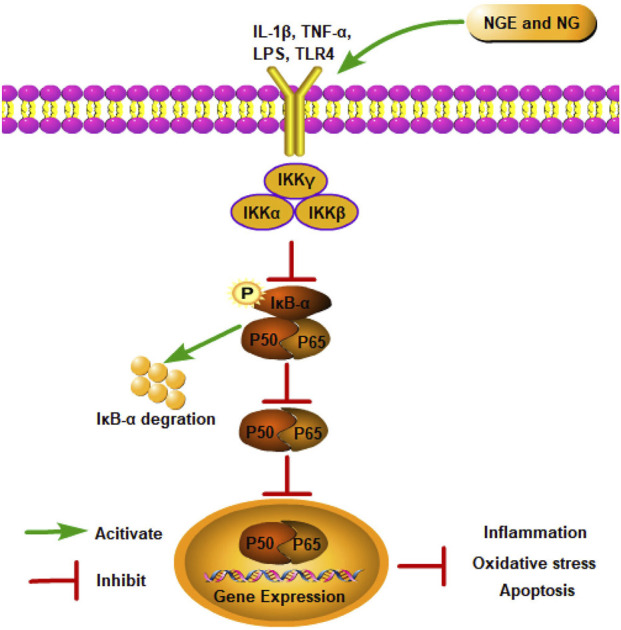
NGE and NG alleviate IRI by inhibiting the NF-κB signaling pathway.

As shown in MCAO/R-induced cerebral IRI rats and kidney IRI rats, NGE treatment reduced oxidative stress and inflammation by inhibiting NF-κB signaling (Dai et al., 2021; Raza et al., 2013). In OGD/R-induced PC12 cells, NG inhibited apoptosis by suppressing NF-κB signaling (Cao et al., 2021). In myocardial IRI, NG alleviated inflammation by suppressing IKK-β/NF-κB (Rani et al., 2013). Additionally, NG showed neuroprotective effects by downregulating excessive free radicals and decreasing inflammation markers *via* inhibition of NF-κB signaling in spinal cord IRI rat models (Cui et al., 2017) (Figure 1).

### 8.2 Nuclear factor erythroid 2-related factor 2 signaling pathway

Nuclear factor erythroid 2-related factor 2 (Nrf2), an essential nuclear transcription factor, serves as a key regulator in mediating the intracellular antioxidant defense system (Zhang et al., 2022). Physiologically, Kelch-like ECH-associated protein-1 (Keap1) degrades Nrf2 *via* ubiquitination complex-dependent mechanisms to maintain Nrf2 dormancy (Sun et al., 2023). As soon as Keap1 is degraded after IRI, its inhibitory effect on Nrf2 is lost. Subsequently, Nrf2 is transferred to the nucleus to activate antioxidant genes (such as heme oxygenase (decycling) 1 (HO-1)) and antioxidant enzymes by forming a heterodimer with the Maf protein (Zhang et al., 2022). In addition to maintaining redox homeostasis, Nrf2 also regulates inflammation and metabolism (Mata and Cadenas, 2021).

**FIGURE 2:**
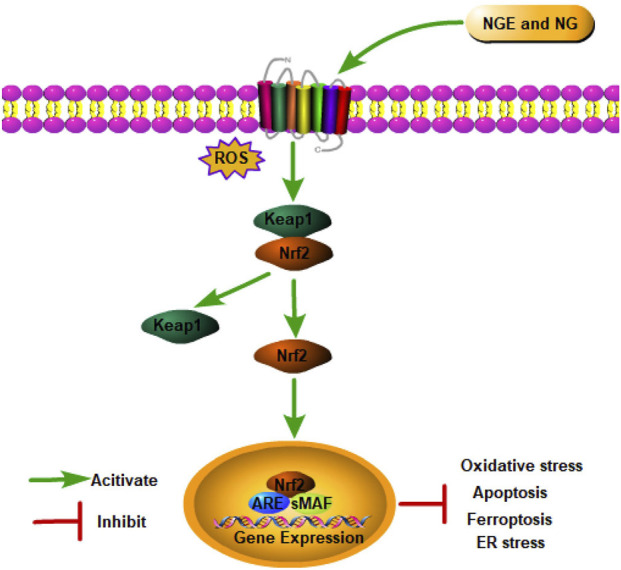
NGE and NG alleviate IRI by activating the Nrf2 signaling pathway.

In myocardial IRI, NGE improved IRI and alleviated oxidative stress by activating Nrf2 signaling (Xu et al., 2021). In cerebral IRI, NGE exerted antioxidant and anti-apoptosis effects by regulating the Nrf2/HO-1 signaling pathway (Wang et al., 2017). NGE was also shown to inhibit ferroptosis by regulating the Nrf2/GPX4 axis, thus alleviating myocardial IRI in rats (Xu et al., 2021). A study conducted on rats with renal IRI found that NG increased Nrf2 expression in kidney tissue, which improved oxidative stress (Amini et al., 2019b). Meanwhile, the Nrf2/HO-1 pathway plays a significant role in ER stress (Yu et al., 2022). In renal IRI, NGE administration attenuated apoptosis and pyroptosis in part by inhibiting ER stress through the activation of Nrf2/HO-1 signaling (Zhang et al., 2022) (Figure 2).

### 8.3 Phosphatidylinositol 3-kinase/AKT signaling pathway

As a bridge between extracellular signals and cellular reactions, the Phosphatidylinositol 3-kinase (PI3K)/AKT signaling pathway regulates cell growth, proliferation, survival, and metabolism (Fukuchi et al., 2004). As PI3K is activated, phosphatidylinositol 3,4,5-triphosphate is formed on the plasma membrane, which changes AKT conformation. Thr308 and Ser473 are the two most significant phosphorylation sites exposed by AKT after it migrates to the cell membrane. Following the phosphorylation of Thr308 and Ser473 by PDK1 and PDK2, AKT is fully activated, regulating cell proliferation, differentiation, and apoptosis (Mayer and Arteaga, 2016). As a critical signaling pathway, PI3K/AKT plays a crucial role in IRI.

**FIGURE 3:**
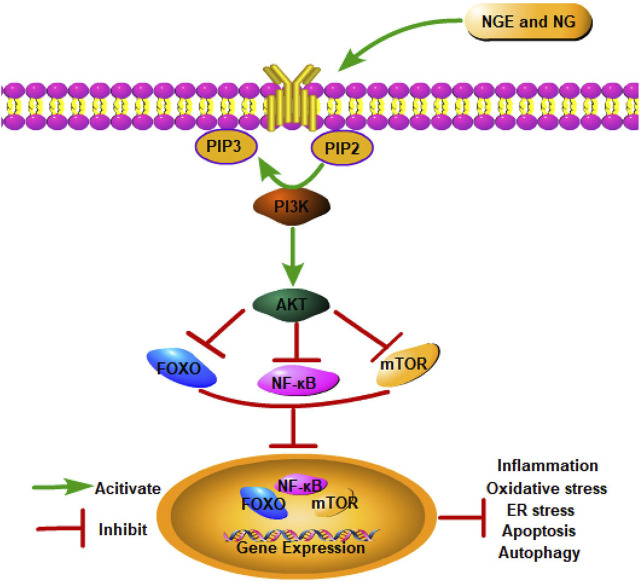
NGE and NG alleviate IRI by activating the PI3K/AKT signaling pathway.

In myocardial IRI, NGE and NG were reported to suppress the IRI-induced cardiac apoptosis, oxidative stress, and inflammation by facilitating the PI3K/AKT signaling pathway (Lan et al., 2021; Li F. et al., 2021). As a result of activating PI3K/AKT signaling, NGE also significantly reduced ER stress in myocardial IRI (Lan et al., 2021). Meanwhile, in myocardial IRI rats, NG inhibited autophagy by activating the PI3K/AKT pathway (Li F. et al., 2021). In cerebral IRI, NG inhibited inflammation and apoptosis *via* activation of the PI3K/AKT pathway (Yang et al., 2020) (Figure 3).

### 8.4 Cyclic guanosine monophosphate–adenosine monophosphate synthase–stimulator of interferon genes signaling pathway

**FIGURE 4:**
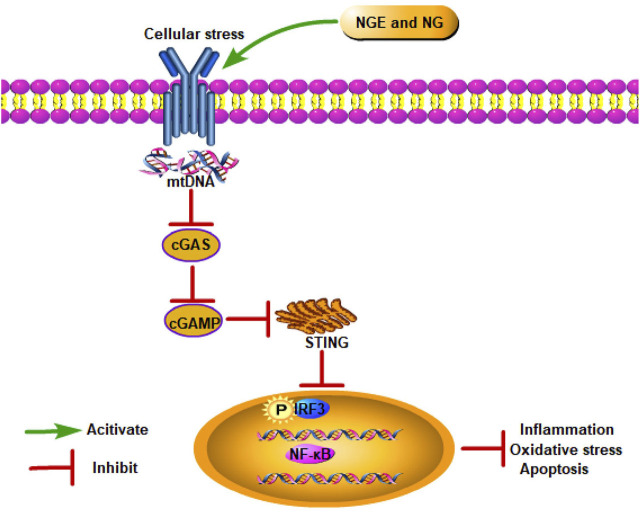
NGE and NG alleviate IRI by inhibiting the cGAS–STING signaling pathway.

In recent studies, the cyclic guanosine monophosphate–adenosine monophosphate synthase–stimulator of interferon genes (cGAS–STING) signaling pathway has been found to be critical to IRI progression (Lei et al., 2022). As a DNA pattern recognition receptor, cGAS recognizes DNA and generates cGAMP to activate STING, subsequently activating NF-κB and IFN regulatory factor (IRF) 3 *via* TANK-binding kinase 1 (TBK1) (Gu et al., 2023). After translocating to the nucleus, NF-κB and IRF3 trigger interferon type I synthesis (IFN-β) and inflammatory cytokines (Wu et al., 2013). In intestinal IRI, NG alleviated inflammation, oxidative stress, and apoptosis by blocking cGAS–STING signaling (Gu et al., 2023) (Figure 4).

### 8.5 Sirtuin 1/Sirtuin 3

Sirtuin (SIRT) 1 is a nicotinamide adenine dinucleotide-dependent histone deacetylase that deacetylates a variety of histones and non-histones (D'Onofrio et al., 2018), modulating downstream targets such as forkhead box O1 (FOXO1) to modulate pathological processes including oxidative stress, inflammation, and apoptosis (Zhao et al., 2023). In myocardial IRI, NG reduced oxidative stress, inflammatory response, and apoptosis by activating the SIRT1 pathway (Liu Y. et al., 2022). In cerebral IRI *in vitro*, NGE also inhibited oxidative stress, inflammation, and apoptosis by enhancing SIRT1/FOXO1 signaling (Zhao et al., 2023). CD38 is a transmembrane glycoprotein that operates *via* receptors and enzymes to regulate inflammation or autoimmune responses (Zeng et al., 2024). A study found that NGE inhibited astrocyte activation in retinal IRI by regulating CD38/SIRT1 signaling, which ultimately reduced retinal inflammation (Zeng et al., 2024). Under stress conditions, SIRT3, a central modulator of mitochondrial protein acetylation, preserves myocardial mitochondrial function and acts as a major downstream component of AMP-activated protein kinase (AMPK) in modulating cardiomyocyte survival (Yu et al., 2019a). A study in myocardial IRI found that NGE decreased mitochondrial oxidative stress and promoted mitochondrial biogenesis by activating the AMPK–SIRT3 pathway (Yu et al., 2019a).

### 8.6 Hypoxia-inducible factor -1α

As a transcriptional regulator of the cellular response to hypoxia, hypoxia-inducible factor (HIF)-1α activates multiple hypoxia-responsive genes to regulate energy metabolism and maintain oxygen homeostasis (Koyasu et al., 2018). Under hypoxic conditions, prolyl hydroxylase activity is inhibited, preventing the degradation of HIF-1α, which is then induced to enter the nucleus and bind to HIF-1β, thus regulating tissue cells to adapt to a hypoxia environment (Shang et al., 2024). It was found that NG normalized HIF-1α levels and alleviated kidney tissue damage in renal IRI (Danis et al., 2024). Meanwhile, in cerebral IRI, NG was shown to inhibit cell apoptosis by modulating the HIF-1α/AKT/mammalian target of the rapamycin (mTOR) signaling pathway (Cao et al., 2021).

## 9 Future recommendations

In future studies, it will be imperative to address the following issues. More research is needed to determine whether NGE and NG are effective and safe for patients. It is also critical to improve NGE and NG bioavailability and pharmacokinetics for clinical applications. In clinical trials, new formulations and techniques such as nanocarriers and crystallization modifications that improve NGE and NG bioavailability and release should also be investigated. At present, NGE and NG are the primary culprits in grapefruit-associated food–drug interactions. It is imperative to explore the mechanisms of drug–drug interactions (augmentation or attenuation) before conducting clinical trials. In addition to determining the correct dose and duration of investigation, it is also imperative to consider the impact of other drugs or supplements that may interact with flavonoids. Meanwhile, research on intestinal microflora would enrich our understanding of the mechanisms behind NGE and NG therapeutic effects in the future. As different flavonoids synergize, combination drug therapy merits further research.

## 10 Discussion

IRI is associated with complex pathological processes, which can be life-threatening, and can be found in various tissues, such as the myocardium, brain, intestines, kidneys, retina, and liver (Lu et al., 2021). Inflammatory responses, oxidative stress, and multiple cell death pathways are involved in IRI pathogenesis and are common across different organs (Li et al., 2025). Furthermore, clinical manifestations of IRI vary considerably across organ systems and are further affected by patients’ underlying conditions, posing difficulties in diagnosis and treatment (Li et al., 2025).

Currently, there are many strategies for preventing and treating IRI. In light of their therapeutic effects and minimal side effects, natural products hold substantial promise in reducing IRI in various organs (Kumar et al., 2025). The natural dietary flavonoids NGE and NG have been shown to exhibit potential therapeutic effects against IRI in preclinical models. In this article, we summarize the recent relevant studies on NGE and NG for treating IRI, covering the myocardium, brain, intestines, kidneys, retina, liver, spinal cord, skeletal muscles, and testicles. Consistent with our review, other flavonoids (quercetin, hesperidin, and luteolin) have also shown protective effects against IRI (Kumar et al., 2025; Xu et al., 2024). Meanwhile, other natural products, such as phenols, terpenoids, and polysaccharides, have been shown to be beneficial for IRI in a variety of organs (Dong et al., 2016; Kumar et al., 2025). In this review, studies focused on the myocardium, brain, kidneys, and intestine. However, research on the retina, liver, spinal cord, skeletal muscles, and testicles is scarce, and further studies are needed.

It has been shown that many signaling pathways (NF-κB, Wnt, Nrf2, and AMPK) are involved in IRI, and they show cross-talk with inflammation, oxidative stress, and programmed cell death, forming a complex network (Zhang M. et al., 2024). In this review, we summarize for the first time the multiple signaling pathways modulated by NGE and NG in organ IRI, which include NF-κB, Nrf2, PI3K/AKT, cGAS–STING, SIRT1/SIRT3, and HIF-1α. Furthermore, we investigate the interactions between these signaling pathways and inflammation, oxidative stress, and programmed cell death. At present, most research remains preliminary, and further studies are required to clarify the cross-talk mechanisms.

Currently, there are few clinical trials of NGE and NG. Studies have found that NGE and NG play a role in hyperlipidemic and overweight patients (Dallas et al., 2014; Salehi et al., 2019). As of now, we have not found any published clinical trials directly related to IRI for NGE and NG. As more preclinical studies report positive results, clinical translation should be accelerated. However, NGE and NG still face many challenges and require well-designed trials utilizing precision medicine approaches and nanotechnology-enabled delivery systems to confirm experimental results in human clinical trials.

## 11 Limitations of the study

Despite this, current research on NGE and NG for the prevention and treatment of IRI has several limitations. First, IRI experiments involve animal and cell experiments. It is difficult to maximize the value of research results because of the differences between experimental animals, modeling methods, and the timing of IRI. Meanwhile, some studies fell short of the high scientific standards. In light of the limited number of studies, we did not exclude any studies based on their quality. Second, the research methods are relatively simple, and most studies measure relevant indicators repeatedly. Although numerous signaling pathways have been identified, there is still no clear understanding of how they interact or regulate one another. Third, we focus exclusively on the biological effects of NGE or NG as there have been very few comparative studies between them. Most animal experiments compare the effects of different dose gradients of NGE and NG longitudinally with modeled groups, but there are few cross-sectional efficacy comparisons with positive control drugs. Finally, only NGE and NG were analyzed for their effects on organ IRI. Other structurally related flavonones, such as hesperidin and nobiletin, were not evaluated. The potential of flavonones to improve organ IRI has not been fully explored.

## 12 Conclusion

During this review, we found that NGE and NG possess antioxidant, anti-inflammatory, anti-apoptotic, anti-ER stress, anti-ferroptosis, anti-pyroptosis, and autophagy regulation properties that protect organs from IRI. Furthermore, NGE and NG reduced organ IRI through certain signaling pathways, including NF-κB, Nrf2, PI3K/AKT, cGAS–STING, SIRT1/SIRT3, and HIF-1α, which were summarized for the first time. Overall, NGE and NG show considerable promise in preclinical organ IRI models. A clear statement must be made that the protective effects of NGE and NG on organ IRI remain at the preclinical trial stage. Further studies are needed to verify their safety and efficacy in clinical settings.

## Glossary

**8-OHdG**: 8-Hydroxydeoxyguanosine
**ASC**: Apoptosis-associated speck-like protein containing a carboxy-terminal
**ATF6**: Activating transcription factor 6
**Bax**: Bcl-2-associated X
**BBB**: Blood–brain barrier
**Bcl-2**: B-cell lymphoma-2
**BDNF**: Brain-derived neurotrophic factor
**CAT**: Catalase
**cGAS**: Cyclic guanosine monophosphate–adenosine monophosphate synthase
**CHOP**: CCAAT/enhancer binding protein homologous protein
**COX**: Cyclooxygenase
**ER**: Endoplasmic reticulum
**FOXO1**: Forkhead box O1
**GPX**: Glutathione peroxidase
**GRP78**: Glucose-regulated protein 78
**GSDMD**: Gasdermin D
**GSH**: Glutathione
**H/R**: Hypoxia–reoxygenation
**HIF**: Hypoxia-inducible factor
**HO-1**: Heme oxygenase (decycling) 1
**IFN**: Interferon
**IL**: Interleukin
**iNOS**: Inducible nitric oxide synthase
IRE1α: Inositol-requiring enzyme 1α
**IRF**: IFN regulatory factor
**IRI**: Ischemia/reperfusion injury
**Keap1**: Kelch-like ECH-associated protein-1
**LC**: Light chain
**MACO/R**: Middle cerebral artery occlusion and reperfusion
MAPK: Mitogen-activated protein kinase
**MDA**: Malondialdehyde
**MPO**: Myeloperoxidase
**NF-κB**: Nuclear factor-κB
**NG**: Naringin
**NGE**: Naringenin
**NLRP3**: Nod-like receptor protein 3
**nNOS**: Neuronal nitric oxide synthase
**NO**: Nitric oxide
**Nrf2**: Nuclear factor erythroid 2-related factor 2
**OGD/R**: Oxygen and glucose deprivation/reperfusion
**PERK**: Protein kinase R-like ER kinase
**PI3K**: Phosphatidylinositol 3-kinase
**ROS**: Reactive oxygen species
**SIRT**: Sirtuin
**SLC7A11**: Solute carrier family 7 member 11
**SOD**: Superoxide dismutase
**STING**: Stimulator of interferon genes
**TBK1**: TANK-binding kinase 1
**TJs**: Tight junctions
**TNF**: Tumor necrosis factor
**UPR**: Unfolded protein response
**ZO-1**: Zonula occludens 1
